# Impressic Acid, a Lupane-Type Triterpenoid from *Acanthopanax koreanum*, Attenuates TNF-α-Induced Endothelial Dysfunction via Activation of eNOS/NO Pathway

**DOI:** 10.3390/ijms20225772

**Published:** 2019-11-16

**Authors:** Sun Woo Jin, Hoa Thi Pham, Jae Ho Choi, Gi Ho Lee, Eun Hee Han, Young Ho Cho, Young Chul Chung, Young Ho Kim, Hye Gwang Jeong

**Affiliations:** 1 College of Pharmacy, Chungnam National University, Daejeon 34134, Korea; mpassword@cnu.ac.kr (S.W.J.); hoapt@cnu.ac.kr (H.T.P.); choijh1@cnu.ac.kr (J.H.C.); ghk1900@cnu.ac.kr (G.H.L.); yhk@cnu.ac.kr (Y.H.K.); 2 Drug & Disease Target Research Team, Division of Bioconvergence Analysis, Korea Basic Science Institute (KBSI), Cheongju 28119, Korea; heh4285@kbsi.re.kr; 3 Department of Pharmaceutics & Biotechnology, College of Medical Engineering, Konyang University, Daejeon 35365, Korea; micael@konyang.ac.kr; 4 Department of Food Science, International University of Korea, Jinju, 52833, Korea; fnjung@hanmail.net

**Keywords:** impressic acid, eNOS, AMPK, CAMKII, Endothelial cells

## Abstract

Atherosclerosis is one of the most reported diseases worldwide, and extensive research and trials are focused on the discovery and utilizing for novel therapeutics. Nitric oxide (NO) is produced mainly by endothelial nitric oxide synthase (eNOS) and it plays a key role in regulating vascular function including systemic blood pressure and vascular inflammation in vascular endothelium. In this study hypothesized that Impressic acid (IPA), a component isolated from *Acanthopanax koreanum*, acts as an enhancer of eNOS activity and NO production. IPA treatment induced eNOS phosphorylation and NO production, which was correlated with eNOS phosphorylation via the activation of JNK1/2, p38 MAPK, AMPK, and CaMKII. In addition, the induction of eNOS phosphorylation by IPA was attenuated by pharmacological inhibitor of MAPKs, AMPK, and CaMKII. Finally, IPA treatment prevented the adhesion of TNF-α-induced monocytes to endothelial cells and suppressed the TNF-α-stimulated ICAM-1 expression via activation of NF-κB, while treatment with L-NAME, the NOS inhibitor, reversed the inhibitory effect of IPA on TNF-α-induced ICAM-1 expression via activation of NF-κB. Taken together, these findings show that IPA protects against TNF-α-induced vascular endothelium dysfunction through attenuation of the NF-κB pathway by activating eNOS/NO pathway in endothelial cells.

## 1. Introduction

Atherosclerosis represents an important health problem and is the leading cause of mortality worldwide. The vascular endothelium plays a vital role in the prevention of atherosclerosis by decreasing oxidative stress, attenuating atherosclerotic events, and preventing vascular inflammatory and adhesion cascades. In fact, vascular endothelial dysfunction leads to impaired nitric oxide (NO) availability, which plays a key role in systemic blood pressure and vascular inflammation [1]. Decreasing NO production in endothelial cells (ECs) is considered a major promoter of atherothrombosis. Hence, the development of strategies to protect and prevent or reduce endothelial dysfunction has become important.

NO, which is a soluble gas continuously produced by the vascular endothelium, has a wide range of biological functions that modulate vasodilatation, blood pressure, mitochondrial respiration, and platelet function [2,3]. In ECs, NO is synthesized from the amino acid l-arginine and molecular oxygen by the constitutively expressed endothelial form of endothelial nitric oxide synthase (eNOS) or NOS3 [4]. NO is produced and released in response to treatment with a variety of different eNOS activators, including thapsigargin, ATP, vascular endothelial growth factor (VEGF), bradykinin, and sphingosine-1-phosphate, as well as by mechanical forces, such as shear stress [5,6]. The phosphorylation of serine and threonine residues of eNOS by protein kinases, such as 5′ AMP-activated protein kinase (AMPK) [7], Ca^2+^ calmodulin-dependent protein kinase II (CaMKII) [8], protein kinase A (PKA) [9], and protein kinase C (PKC) [10] is believed to be important for regulating the activity of eNOS in ECs [11]. It has also been reported that Akt is related to VEGF-dependent phosphorylation of Ser1177 of human eNOS (equivalent to Ser1179 in bovine eNOS) increases eNOS activity and enhances the production of NO [12,13,14,15].

Impressic acid (IPA; 3α-11α-dihydroxylup-20(29)-en-28-oic acid) is a lupane-type triterpenoid that was isolated for the first time from *Schefflera impressa* [16]. In Korea, it is found in the roots [17] and leaves [18] of *Acanthopanax koreanum*, which has been used as a folk medicine for rheumatism, hepatitis, type 2 diabetes, and inflammatory disorders. IPA has been reported to possess the ability to downregulate matrix metalloproteinase-13 activity and protect against cartilage destruction [19]. IPA also inhibits tumor necrosis factor (TNF)-α-induced nuclear factor-κB (NF-κB) activity and can upregulate the transcriptional activity of peroxisome proliferator-activated receptor γ (PPARγ) [20]. Although IPA possesses important biological properties, the effects of IPA on eNOS activity and NO production in human ECs have not been previously reported. In the present study, we investigated the vascular protective effect of IPA by elucidating the molecular mechanism involved in the IPA-dependent increase in eNOS activity using cultured EA.hy926 human vascular ECs.

## 2. Results

### 2.1. Cytotoxicity of IPA in EA.hy926 Cells

**Figure 1:**
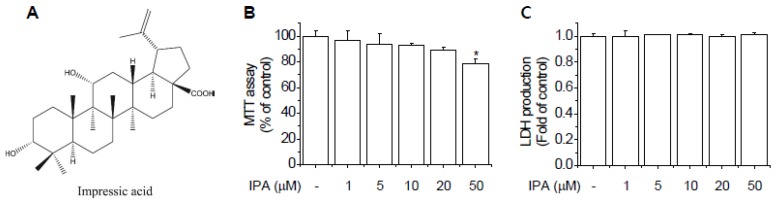
The effects of impressic acid (IPA) on the cytotoxicity of EA.hy926 cells. (**A**) Chemical structure of IPA. (**B**,**C**) EA.hy926 cells were treated with 1, 5, 10, 20, and 50 μM IPA for 24 h. Cytotoxicity was assessed by 3-(4,5-dimethylthiazol-2-yl)-2,5-diphenyltetrazolium bromide (MTT) (**B**) and LDH (**C**) assays. Data are means ± SD of three independent experiments. * *p* < 0.05 compared with control.

The chemical structure of IPA is shown in Figure 1A. The cytotoxicity of IPA to EA.hy926 cells was determined using 3-(4,5-dimethylthiazol-2-yl)-2,5-diphenyltetrazolium bromide (MTT) and LDH assays. As shown in Figure 1B,C, IPA did not cause significant cytotoxicity at concentrations less than 50 μM. Thus, we used a range of 1–20 μM IPA in subsequent experiments.

### 2.2. IPA Increases eNOS Phosphorylation and NO Production in Endothelial Cells

**Figure 2:**
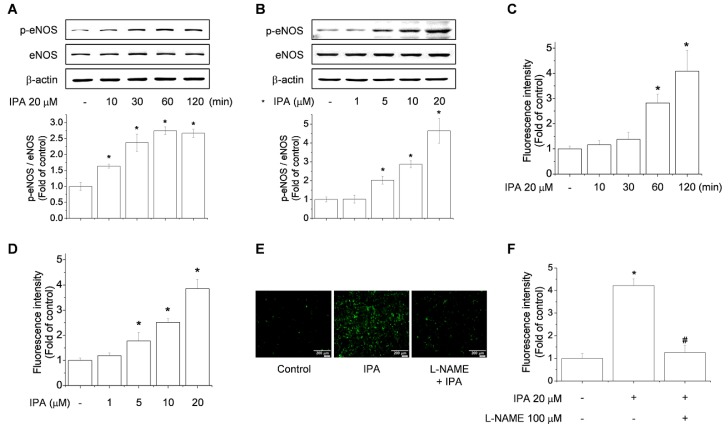
IPA treatment induces endothelial nitric oxide synthase (eNOS) activity and NO production. EA.hy926 cells were treated with 20 μM IPA for 10, 30, 60, and 120 min (**A**,**C**) or 1, 5, 10, and 20 μM IPA for 60 min (**B**,**D**), and assessed by western blotting (**A**,**B**) or measured using the NO-specific fluorescent dye 4,5-Diaminofluorescein diacetate (DAF-2 DA) at 495/515 nm (**C**,**D**). Cells were pretreated with 100 μM l-NAME (NOS inhibitor) for 60 min before treatment with 20 μM IPA for 60 min at 37 °C, and NO production was visualized and measured using the NO-specific fluorescent dye DAF-2 DA at 495/515 nm (**E**,**F**). Data are means ± SD of three independent experiments. * *p* < 0.05 compared with control. # *p* < 0.05 compared with IPA treatment.

eNOS activity is regulated by the phosphorylation/dephosphorylation state of the enzyme. In particular, phosphorylation of eNOS at Ser1177 is pivotal in regulating NO generation [21]. As shown in Figure 2A, IPA treatment upregulated phosphorylation of eNOS-Ser1177 as early as 10 min post-stimulation and this persisted until 120 min post-stimulation. When EA.hy926 and human umbilical vein endothelial cells (HUVECs) were also stimulated with various concentrations of IPA, we found that eNOS phosphorylation was significantly increased in response to 5 μM IPA, and a maximal induction was observed at 20 μM (Figure 2B; Figure S1A). Similar findings were observed in terms of NO production under IPA treatment conditions (Figure 2C,D and Figure S1B). NO production stimulated by IPA was inhibited by the NOS inhibitor, L-NAME (Figure 2E,F and Figure S1C). Taken together, IPA induces eNOS activity and concomitant NO production in a time- and concentration-dependent manner in endothelial cells.

### 2.3. AMPK and CaMKII Are Required for IPA-Induced eNOS Phosphorylation and NO Production

**Figure 3:**
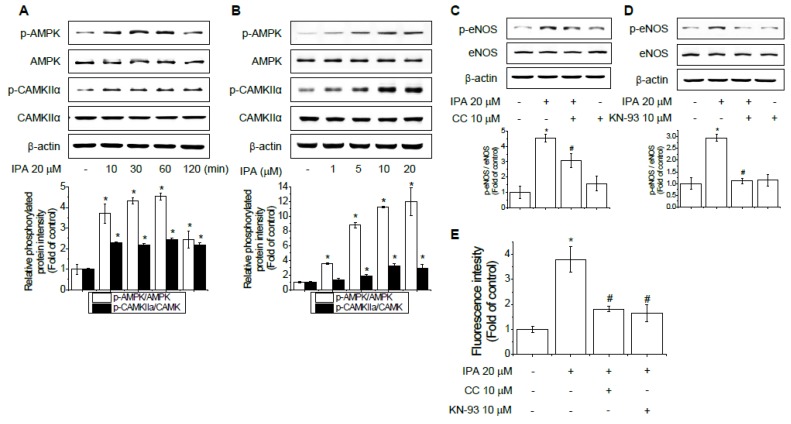
Phosphorylation of eNOS induced by IPA is mediated by 5′ AMP-activated protein kinase (AMPK) and Ca^2+^ calmodulin-dependent protein kinase II (CaMKII). Immunoblots of EA.hy926 cell lysates treated with 20 μM IPA for 10, 30, 60, and 120 min (**A**) or with different concentrations of IPA (1, 5, 10, and 20 μM) for 60 min (**B**). EA.hy926 cells were treated with 10 μM of the AMPK inhibitor compound C (**C**) or 10 μM of the CaMKII inhibitor KN-93 (**D**) for 1 h, followed by incubation with or without 20 μM IPA for an additional hour. NO production was analyzed with the NO-specific fluorescent dye DAF-2 DA kit at 495/515 nm (**E**). Data are means ± SD of three independent experiments. * *p* < 0.05 compared with control. # *p* < 0.05 compared with IPA treatment.

AMPK is a sensor of cellular energy state and a regulator of cellular homeostasis [22,23]. Previously, AMPK has been reported to activate eNOS at Ser1177 [23,24,25]. CaMKII also regulates eNOS expression by altering the level of eNOS-Ser117 phosphorylation and NO production in ECs [26,27]. Western blotting indicated that IPA treatment increased AMPK and CaMKII phosphorylation in a time- and concentration-dependent manner in EA.hy926 cells (Figure 3A,B).

The AMPK and CaMKII inhibitors compound C and KN-93, respectively, were used to determine whether AMPK and CaMKII are required for IPA-induced eNOS-Ser1177 phosphorylation and NO production. Interestingly, eNOS-Ser1177 phosphorylation and NO production in ECs were attenuated by IPA and compound C or KN-93 treatment (Figure 3C–E). These data suggest that eNOS activity and NO production promoted by IPA-induced phosphorylation are dependent on AMPK and CaMKII signaling.

### 2.4. Role of Akt and MAPKs in IPA-Induced eNOS Phosphorylation and NO Production

**Figure 4:**
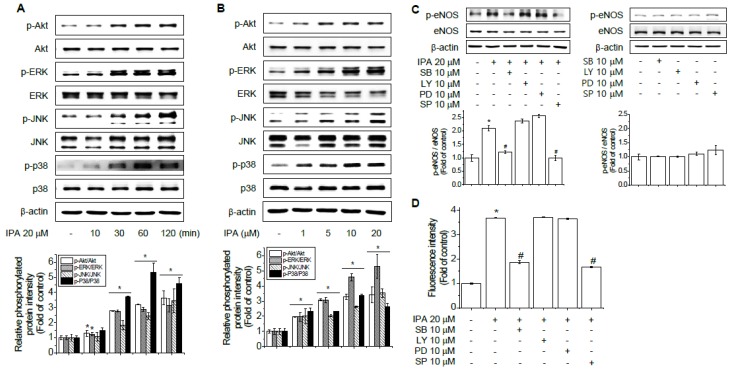
Akt and MAPKs related to eNOS activity and NO production in EA.hy926 cells induced by IPA. Immunoblots of EA.hy926 cell lysates treated with 20 μM IPA for 10, 30, 60, and 120 min (**A**) or with different concentrations of IPA (1, 5, 10, and 20 μM) for 60 min (**B**). EA.hy926 cells were treated with PD98059 (ERK1/2 inhibitor), SB203580 (p38 inhibitor), SP600125 (JNK1/2 inhibitor), and LY (PI3K/Akt inhibitor) for 1 h, followed by incubation with or without 20 μM IPA for an additional hour (**C**). NO production was analyzed with the NO-specific fluorescent dye DAF-2 DA kit at 495/515 nm (**D**). Data are means ± SD of three independent experiments. * *p* < 0.05 compared with control. # *p* < 0.05 compared with IPA treatment.

Recent data has shown that direct phosphorylation of eNOS can occur via the PI3K pathway by activating Akt, which reduces the enzyme’s calcium requirement and results in increased production of NO [28,29]. P38 MAPK (p38), ERK, and JNK have also been reported to be involved in vascular relaxation and NO production [30,31]. Therefore, we examined the activity of Akt, ERK, p38, and JNK in IPA-treated EA.hy926 cells. Western blot analysis indicated that treatment of EA.hy926 cells with IPA resulted in a sustained phosphorylation of Akt, ERK, JNK, and p38 in a time- and concentration-dependent manner (Figure 4A,B). To further elucidate whether activation of Akt and MAPKs is required for eNOS phosphorylation, we used LY-294002 (inhibitor of PI3K, the upstream activator of Akt), PD98059 (ERK1/2 inhibitor), SB203580 (p38 inhibitor), and SP600125 (JNK1/2 inhibitor) prior to stimulation with IPA. Treatment of ECs with these inhibitors did not affect basal levels of eNOS. On the other hand, pharmacological inhibition of p38 and JNK1/2 significantly reduced IPA-mediated induction of eNOS and NO production while inhibitors of Akt and ERK1/2 had no effect (Figure 4C,D). These data indicate that IPA stimulates the phosphorylation of eNOS in EA.hy926 cells is independent of Akt, ERK1/2, yet p38 and JNK1/2 were necessary for eNOS activation and NO production.

### 2.5. Effects of IPA on the Inhibition of NF-κB Activation, ICAM-1 Expression, and Monocyte Adhesion in ECs

**Figure 5:**
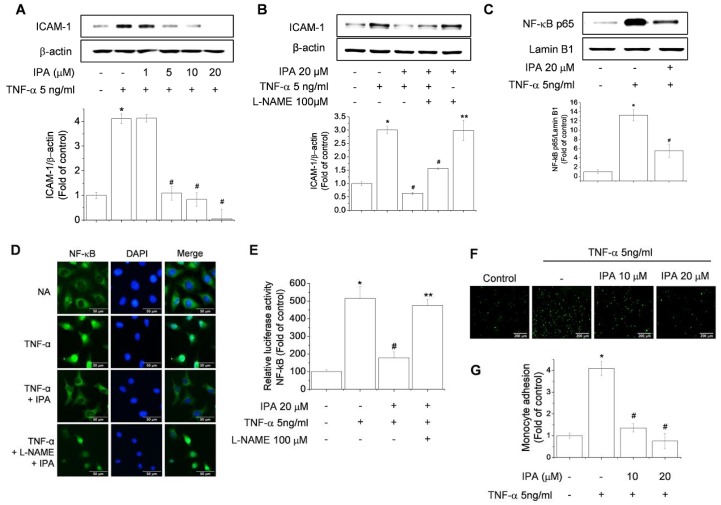
IPA treatment decreases the expression of adhesion molecules and inhibits the NF-κB pathway. EA.hy926 cells were treated with different concentrations of IPA (1, 5, 10, and 20 μM) for 1 h, followed by incubation with 5 ng/mL TNF-α for 24 h (**A**). Cells were pretreated with 100 μM of the NOS inhibitor L-NAME for 1 h, and then treated with 20 μM IPA for an additional hour, followed by incubation with 5 ng/mL TNF-α for 24 h (**B**). Cells were treated with 20 μM for 1 h, followed by incubation with 5 ng/mL TNF-α for 6 h (**C**). Cell lysates were analyzed by western blotting using specific antibodies. The inhibitory effect of IPA on TNF-α-induced NF-κB activation blocked by L-NAME was assessed by reporter assay and immunofluorescence (**D**,**E**). EA.hy926 cells were pretreated with 10 or 20 μM IPA for 1 h and TNF-α was added at 5 ng/mL for an additional 6 h. EA.hy926 cells were co-cultured with U937 monocytes for 1 h, and the adherence of endothelial cells to monocytes was assessed by fluorescence microscopy (**F**,**G**). Data are means ± SD of three independent experiments. * *p* < 0.05 compared with control. # *p* < 0.05 compared with IPA treatment. ** *p* < 0.05 compared with IPA and TNF-α treatment.

The transcription factor NF-κB plays an essential role in inflammation in ECs [32]. Furthermore, NO derived from eNOS is known to exert direct anti-inflammatory effects [33]. Thus, with respect to the emerging role of inflammatory responses in the development of endothelial dysfunction and its attenuation due to NO release, we examined if IPA had an effect on TNF-α-induced NF-κB signaling, or on the expression of intercellular cell adhesion molecule (ICAM) in EA.hy926 cells. As expected, treatment of EA.hy926 cells with TNF-α, an NF-κB-activating inflammatory stimulus, increased the expression of ICAM and p65, while this increase was significantly suppressed by treatment with IPA (Figure 5A,C). In addition, treatment with L-NAME, the NOS inhibitor, markedly reversed the inhibitory effect of IPA on TNF-α-induced ICAM expression (Figure 5B). Fluorescence microscopy images showed that in untreated cells, the NF-κB p65 protein is maintained in an inactive state in the cytoplasm, whereas stimulation of cells with TNF-α resulted in the nuclear localization of p65 NF-κB. However, cells treated with 20 μM IPA exhibited diminished staining of nuclear p65 (Figure 5D). In addition, IPA treatment decreased NF-κB-dependent promoter activity (Figure 5E), while treatment with L-NAME significantly reversed the inhibitory effect of IPA on TNF-α-induced activation and translocation of NF-κB (Figure 5D,E). At 10–20 μM, IPA also substantially inhibited the adhesion of U937 monocytes to ECs (Figure 5F,G). Emerging evidence suggests that IPA may help attenuate endothelial dysfunction by opposing the atherogenic actions of pro-inflammatory cytokines.

## 3. Discussion

Previous studies have demonstrated the anti-inflammatory effects of IPA, i.e., a significant reduction in the inhibitory activity of the nuclear factor of activated T-cells and reduction of the production of pro-inflammatory cytokines, including TNF-α, interleukin (IL)-6, and IL-12 p40 by lipopolysaccharide stimulation in bone marrow-derived dendritic cells [18,34]. IPA has also been shown to up-regulate PPARα1, PPARα2, and sterol regulatory element-binding protein 2 (SREBP-2), and suppress the expression of insulin-induced gene 2 (Insig-2) [20]. However, there have been few pharmacological studies of IPA focusing on vascular protection, and the mechanism has not been fully elucidated. Our study is the first to demonstrate that IPA rapidly stimulates eNOS phosphorylation on Ser1177 in EA.hy926 cells, resulting in an acute increase in NO production. Furthermore, IPA stimulated PI3K/Akt, MAPK, CaMKII, as well as AMPK activation, which play an important role in mediating IPA-stimulated eNOS activation.

Protein–protein interactions play an important role in eNOS activity, which is tightly controlled by co- and post-translational phosphorylation and lipid modifications. Treatment of ECs with VEGF, hypoxia, PPAR agonists, and adiponectin can stimulate AMPK-dependent phosphorylation of eNOS-Ser1177 [35]. Transfection of human ECV304 ECs with a constitutively active form of AMPK was sufficient to increase eNOS phosphorylation, indicating that AMPK also functions as an eNOS activator [36]. In the present study, the tested concentrations of IPA were based on the non-cytotoxic concentration range, which is consistent with previous in vitro studies using IPA [19,20]. IPA induced AMPK phosphorylation in a concentration- and time-dependent manner. In addition, the AMPK inhibitor reduced AMPK and eNOS activity, as well as NO production, indicating that IPA induces AMPK-dependent eNOS activation.

Ca^2+^- CaMKII signaling is reported to play a crucial role in eNOS activation and NO production [37]. In the present study, phosphorylation of CaMKII was positively regulated by IPA. Furthermore, the CaMKII inhibitor, KN-93, significantly inhibited IPA-induced phosphorylation of eNOS. Interestingly, in our previous study, betulinic acid ((3β)-3-hydroxy-lup-20(29)-en-28-oic acid), which has a chemical structure similar to IPA, also showed AMPK- and CaMKII-dependent eNOS activation [38]. Though IPA and betulinic acid originate from different plant families, the high chemical similarity may explain the molecular mechanism of IPA-induced eNOS activation.

The intracellular signaling kinase, Akt, which is phosphorylated in response to increased mechanical force in cultured ECs, also induces phosphorylation of eNOS [39]. Inhibition of the PI3K/Akt pathway or mutation of the Akt site attenuates the activation of eNOS [28]. The PI3K/Akt/eNOS signaling pathway is critical for maintenance of endothelial vascular tone and integrity [40,41]. MAPKs, including ERK, p38, and JNK, are important signaling pathways that are involved in cell metabolism, growth, and expression, and the eNOS pathway is often also involved [42,43,44]. In the present study, we observed that IPA increased the phosphorylation of Akt and MAPKs (p38 MAPK, JNK, and ERK1/2) in a time and concentration-dependent manner. However, the inhibition of Akt and ERK1/2 did not suppress IPA-induced phosphorylation of eNOS in EA.hy926 cells as well as NO production while JNK1/2 and p38 abolished strongly eNOS activity and NO production in EA.hy926. These results suggest that IPA-induced eNOS activity is involved in the p38 and JNK1/2 pathways.

Activation of NF-κB causes transcriptional activation of genes encoding adhesion molecules such as ICAM-1 and vascular cell adhesion molecule-1 (VCAM-1), which are responsible for monocyte adhesion and are involved in the pathogenesis of a variety of vascular inflammatory diseases [29]. Recently, a number of studies have shown that promotion of eNOS activation and NO production in the endothelium contributes to NF-κB inactivation, and attenuates atherosclerosis [45,46]. Moreover, the ability of NO to inhibit the expression of endothelial–leukocyte adhesion molecules and certain pro-inflammatory cytokines makes it a potentially important regulator of inflammatory trafficking within the vessel wall [47]. Bath and co-workers reported that NO inhibits monocyte adhesion to the endothelium in vitro, without altering expression of CDl1b/CD18, one of the cognate ligands on monocytes for endothelial leukocyte adhesion molecules [48]. Consistent with a previous report [49], IPA significantly reduced TNF-α-induced ICAM-1 expression, NF-κB activation, and monocyte adhesion. However, the inhibitor L-NAME strongly blocked the IPA-stimulated inhibition of cytokine-induced ICAM-1 expression and NF-κB activation. These results suggest that the inhibition of ICAM-1 expression and NF-κB activation might directly mediate the IPA-dependent stimulation of NO production. In conclusion, IPA induces eNOS activation via the AMPK, CaMKII, p38 and JNK1/2 pathways, and IPA-induced NO generation inhibits vascular inflammation by downregulating ICAM-1 expression and NF-κB activation. Moreover, this study provides important insights into the beneficial effects of IPA on protection of the vasculature from EC dysfunction and may contribute to the development of new therapeutic drugs for vascular diseases.

## 4. Materials and Methods

### 4.1. Chemicals and Reagents

IPA was provided by Dr. Young-Ho Kim (Chungnam National University, Daejeon, Korea). Dulbecco’s modified Eagle’s medium (DMEM), fetal bovine serum (FBS), and trypsin-EDTA were obtained from Gibco-BRL (Grand Island, NY, USA). KN-93 and TNF-α were purchased from Sigma-Aldrich (St. Louis, MO, USA). LY294002, PD98059, SB203580, SP600125, compound C, and L-NAME were purchased from Calbiochem (La Jolla, CA, USA). 4,5-Diaminofluorescein diacetate (DAF-2 DA) was obtained from Invitrogen (Carlsbad, CA, USA). Antibodies against phospho-AMPKα, phospho-eNOS, eNOS, phospho-CaMKII, phospho-Akt, Akt, phospho- extracellular signal-regulated kinase (ERK), ERK, phospho-c-Jun N-terminal kinase (JNK), JNK, phospho-p38, and p38 were purchased from Cell Signaling Technology (Danvers, MA, USA). Antibodies against NF-κB, AMPKα and β-actin were obtained from Santa Cruz Biotechnology (Santa Cruz, CA, USA). The tetrazole 3-(4,5-dimethylthiazol-2-yl)-2,5-diphenyltetrazolium bromide (MTT) was acquired from USB Corporation (Cleveland, OH, USA). The cytotoxicity detection kit was obtained from Roche Applied Science (Indianapolis, IN, USA). The enhanced chemiluminescence (ECL) system was obtained from BioFact (Daejeon, Korea) and polyvinylidene difluoride (PVDF) membranes were purchased from Amersham Pharmacia Biotech (Uppsala, Sweden). All other chemicals were of the highest commercial grade available.

### 4.2. Cell Culture and Treatment

EA.hy926 cells were obtained from the American Type Culture Collection (Bethesda, MD, USA) and cultured in DMEM supplemented with 10% FBS, 100 U/mL penicillin, and 100 μg/mL streptomycin (HyClone, Logan, UT, USA). HUVECs were obtained from Lonza (Walkersville, MD, USA) and cultured in Endothelial Growth Medium 2 (Lonza). The cells were incubated at 37 °C in a humidified incubator containing 5% CO2. In all in vitro experiments, cells were used at passages 3–10. IPA was dissolved in dimethylsulfoxide (DMSO) and stored at −20 °C until use. The control cells were treated with DMSO alone and the final concentration of DMSO was kept at <0.2%.

### 4.3. Measurement of Cell Cytotoxicity

Conventional MTT reduction and LDH assays were used to determine the toxicity of IPA to EA.hy926. Cells were seeded in DMEM medium containing 10% FBS in 48-well plates at 37 °C for 24 h. The following day, cells were treated with different concentrations of IPA and the plates were incubated at 37 °C for 24 h. MTT solution was added, followed by incubation for 30 min, and formazan crystals were solubilized by adding DMSO. The absorbance at 550 nm was measured using a BioTek Synergy HT microplate reader (BioTek Instruments, Winooski, VT, USA). The media were collected to perform a lactate dehydrogenase assay and the absorbance was measured at 490 nm with a BioTek Synergy HT microplate reader (BioTek Instruments). Cell viability (%) and cytotoxicity (fold-change) were quantified based on the absorbance of treated cells relative to control (exposed to DMSO alone).

### 4.4. Protein Extraction and Western Blotting

Cell lysates were prepared in lysis buffer (120 mM NaCl, 40 mM Tris (pH 8), and 0.1% Nonidet P-40) on ice for 30 min and centrifuged at 13,000 rpm for 15 min. The supernatant was collected as the source of sample protein and concentrations were determined at 595 nm using a protein assay kit (Pro-Measure; Intron Biotechnology, Seongnam, Korea). Equal amounts of total cellular protein were separated by 10% sodium dodecyl sulfate polyacrylamide gel electrophoresis and transferred onto PVDF membranes. After blocking with 5% skim milk for 1 h, blots were incubated with primary antibodies overnight, followed by incubation with a horseradish peroxidase-conjugated secondary antibody. The protein bands were visualized using the ECL western blot detection system. ImageJ software (NIH, Bethesda, MD, USA) was used to calculate the integrated OD for the protein band and the values were normalized to an internal control.

### 4.5. Quantification of NO

The production of NO was measured using the complimentary fluorescent NO indicator probe, DAF-2 DA (Invitrogen). DAF-2 DA is cell-permeable, passively diffuses across cell membranes and is commonly used to detect intracellular NO levels in living cells [50,51,52]. EA.hy926 cells were cultured to 90% confluence in 48-well plates and serum-starved overnight. Before IPA treatment, the cells were incubated with DAF-2 DA at a final concentration of 5 µM for 30 min at 37 °C, rinsed three times with fresh media to remove excess probe, and incubated for an additional 15 min at room temperature to allow for complete de-esterification. For inhibition experiments, the NOS, AMPK, and CAMK inhibitors, l-NAME (100 µM), compound C (10 µM), and KN-93 (10 µM), respectively, were added 30 min before loading with DAF-2 DA. The fluorescence was measured using a fluorescence spectrophotometer, the BioTek Synergy HT microplate reader (BioTek Instruments), at 495/515 nm and selected cells were captured with an EVOS fluorescence microscope (Life Technologies, Carlsbad, CA, USA).

### 4.6. Transient Transfection and Luciferase Reporter Assay

NF-κB reporter construct was purchased from BPS Bioscience, Inc. (Cornerstone Court West, San Diego, CA, USA). Briefly, EA.hy926 cells were seeded in 48-well plates for 24 h and transiently transfected with 500 ng/well of luciferase reporter construct and 50 ng/well of internal control plasmid of the pCMV-β-galactosidase reporter plasmid using Lipofectamine 2000 reagent, according to the manufacturer’s instructions (Invitrogen). NF-κB-promoter activity was determined using a dual-luciferase reporter assay system (Promega, Madison, WI, USA).

### 4.7. Immunofluorescence Staining

EA.hy926 cells were grown on glass coverslips to 60–80% confluence. The cells were fixed in 4% paraformaldehyde for 20 min at room temperature and blocked with 5% serum for 30 min. After five washes with phosphate-buffered saline (PBS) with 0.2% Tween, glass coverslips were immersed in 0.2% Triton X-100 in PBS for 10 min. Thereafter, EA.hy926 cells were incubated with a primary antibody against NF-κB (1:200) at 4 °C overnight. After washing, cells were incubated with Alexa Fluor 532-conjugated secondary (1:500; Molecular Probes, Eugene, OR, USA) for 2 h at room temperature. Cellular nuclei were stained with 4′,6-diamidino-2-phenylindole (DAPI; 1:1,000 dilution).

### 4.8. Cell–Cell Adhesion Assay

Cell–cell adhesion experiments were conducted using EA.hy926 and U937 human leukemic-monocyte lymphoma cells under normal conditions and following stimulation with TNF-α. Confluent EA.hy926 cells in 48-well plates were incubated with IPA for 1 h and stimulated with TNF-α for 6 h. U937 cells were fluorescently labeled using calcein-AM (BD Biosciences, Bedford, MA, USA) for 30 min at 37 °C and washed twice with DMEM medium. EA.hy926 and labeled U937 cells (2 × 105 cells/well) were co-incubated for 30 min at 37 °C in a CO_2_ incubator for 1 h. The non-adherent cells were removed by washing twice with PBS. Monocyte cells bound to EA.hy926 cells were visualized by fluorescence microscopy (EVOS). Spectrofluorometric quantification was conducted using a Varioskan spectrofluorimetric microplate reader (Thermo Electron Co., Waltham, MA, USA) at emission and excitation wavelengths of 485 and 535 nm, respectively.

### 4.9. Statistical Analysis

All experiments were repeated at least three times. Results are reported as means ± SD. One-way analysis of variance (ANOVA) was used to determine the significance of differences between treatment groups. The Newman–Keuls test was used for multi-group comparisons. Statistical significance was defined as *p* <0.05.

## Supplementary Materials

Supplementary materials can be found at https://www.mdpi.com/1422-0067/20/22/5772/s1.

## Abbreviations

AMPK	AMP-activated protein kinase
CaM	Calmodulin
CaMKII	Ca^2+^/calmodulin-dependent kinase II
CC	Compound C
eNOS	Endothelial nitric oxide synthase
IPA	Impressic acid
MAPK	Mitogen-activated protein kinase
NO	Nitric oxide
