# Supplementary material for: Impressic Acid, a Lupane-Type Triterpenoid from Acanthopanax koreanum, Attenuates TNF-α-Induced Endothelial Dysfunction via Activation of eNOS/NO Pathway
Source: Int J Mol Sci. 2019 Nov 16;20(22):5772. doi: 10.3390/ijms20225772 (PMC6888592; doi:10.3390/ijms20225772)
Supplement: Supplementary file 1 [file ijms-20-05772-s001.pdf]

Supplementary figure

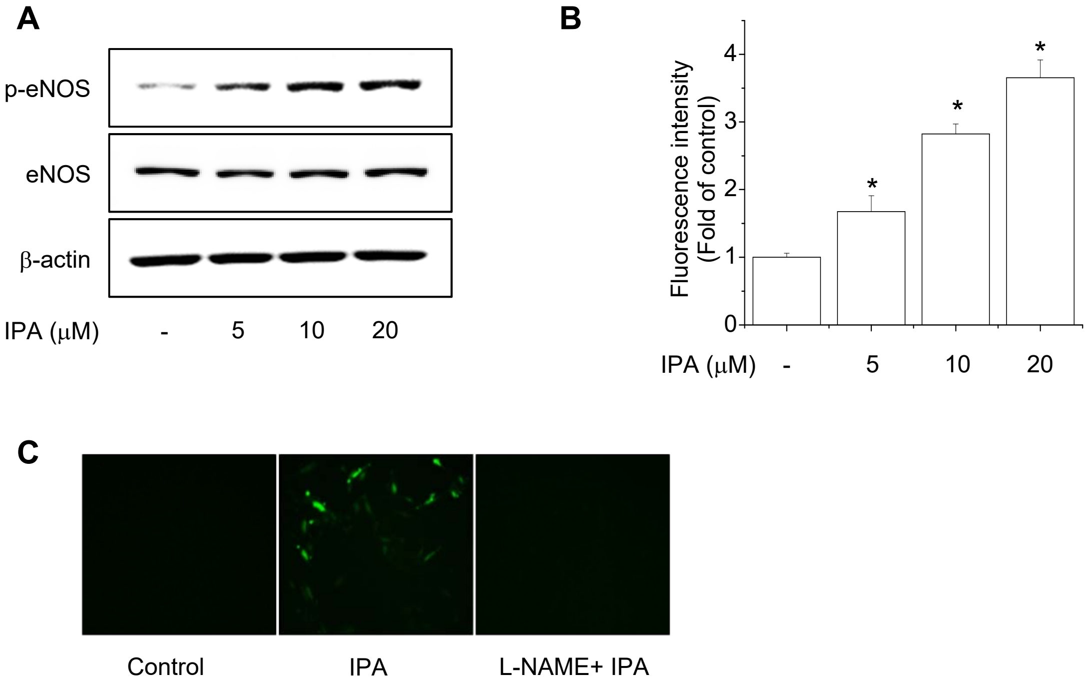

Figure S1. IPA treatment induces eNOS activity and NO production in HUVECs. HUVECs were treated with 5, 10, and 20 μM IPA for 60 min (A, B), and assessed by western blotting (A) or measured using the NO-specific fluorescent dye DAF-2 DA at 495/515 nm (B). Cells were pretreated with 100 μM of the NOS inhibitor L-NAME for 1 h, and then treated with 20 μM IPA for an additional hour at 37°C, and NO production was visualized and measured using the NO-specific fluorescent dye DAF-2 DA at 495/515 nm (C). *$p < 0.05$ compared with control.
